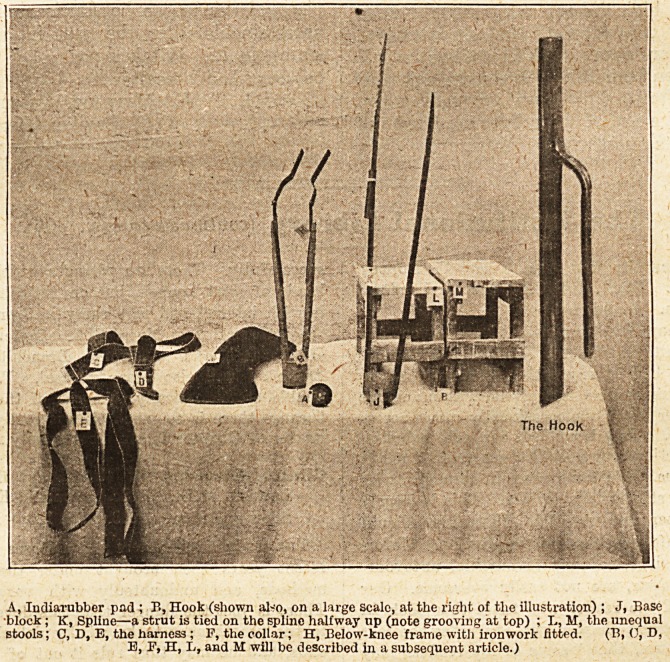# As Made at the Pavilion General Hospital, Brighton

**Published:** 1918-07-06

**Authors:** G. T. K. Maurice

**Affiliations:** Colonel A.M.S., O.C. Pavilion General Hospital.


### TEMPORARY ARTIFICIAL LEGS

### As Made at the Pavilion General Hospital, Brighton.

The Editor of The Hospital has complimented me by an invitation to contribute on the subject that heads this article, and has made flattering references in his letter to the work that is done at the Pavilion. All concerned thank him for the courtesy done to us.

There is no claim made that any of this work is an original invention of the Pavilion Hospital, though many modifications of design and method have been evolved in the course of practice.

The history of the department is: Dr. D. F. Martin, Ambulance de l'Océan, La Panne, who has written in French a book on the subject of temporary limbs which was published by Masson et Cie., 120 Boulevard St. Germain, came to Brighton at the instance of Mrs. Bromley-Davenport and taught his method to workers of the Hove War Hospital Supply Depot. Of these workers one, Lady Shiffner, an American by birth, developed great aptitude. Lady Shiffner came to the Pavilion Hospital buildings, and there organised the Plaister Room, which, from a small beginning under the fostering care of Col. R. E. S. Davis, I.M.S., who was then commanding the hospital, and Major W. A. Chapple, M.P., R.A.M.C., senior operating surgeon, has in the course of a few months grown to its present state of large activity and usefulness. Formerly the output was about a leg a day; now eight and nine legs are finished on many days.

The Plaister Room is a spacious apartment of the Prince Regent's Palace. It is said that at one time it was a queen's chamber, but now it is a workshop fitted with two lathes, a large carpenter's bench and a smaller one, and an assortment of tools for working wood and iron, all supplied free of charge by the generous lender, who has asked me to obliterate her name. The other furnishings are a few tables, chairs, cupboards, plaister-bins, and utensils for liquid plaister and water and a medley of miscellaneous material for the work.

A, Indiarubber pad; B, Hook (shown also, on a large scale, at the right of the illustration); J, Base block; K, Spline—a strut is tied on the spline halfway up (note grooving at top); L, M, the unequal stools; C, D, E, the harness; F, the collar; H, Below-knee frame with ironwork fitted. (B, C, D, E, F, H, L, and M will be described in a subsequent article.)

The idea is to supply a very cheap and comfortable temporary artificial limb which shall enable a man to get about without a crutch before he is ready for a permanent and elaborate artificial leg, and which, by its pressure and the effects of use, shall cause that shrinkage and moulding of the stump which always takes place, and continues for some time, after an amputation and the use of an artificial leg.

The amount of shrinkage and the time during which it continues varies vastly in different cases, but is inevitable in all. If an artificial limb is supplied and fitted only a few weeks after recovery and healing the costly apparatus soon ceases to fit, and alterations or renewals more satisfactory to the instrument-maker than to the patient or the taxpayer are required. We believe here that if every man wore a cheap temporary artificial limb for the first six months or so after his stump was fit to bear one, that the taxpayers would be saved hundreds of thousands of pounds, and, even more important, thousands of men who now go into civil life with a costly leg and are deeply chagrined to find after a few weeks that they have to return it and get another fitted, with consequent interruption of their work and ways, would be saved the disappointment.

The making is a simple matter, but it requires practice, some ingenuity, and the knack of using the fingers deftly. The legs are divisible into two main classes—those for above-the-knee stumps and those for below-the-knee stumps, but, as will be seen, there are no standard patterns. Each leg is a separate work of art; each leg is a fit for its owner, and not a fit for any other person.

Since it is simpler to make and fix a leg to a

**TEMPORARY ARTIFICIAL LEGS**—*(continued).*

thigh stump than to a leg stump, that making and fitting shall be firstly described.

Above-the-Knee Legs.

The man applying for a leg comes to the Plaister Room and has his name and the day and time appointed for the making of the cast entered in a book. He then goes to the mechanic. This man is a wounded soldier, a patient in the hospital, and the mechanic finds by measurement the length of the wooden part of the leg.

The frame on to which the plaister bucket will be fitted consists of five pieces (see illustration, p. 353)—a circular indiarubber pad (A) such as is often worn on the heels of boots, a base block (G), two splints (K), and a strut. The indiarubber pad needs no description. The base block is a piece of wood, circular in horizontal section, about 2½ inches high, about 3½ inches across the bottom. Any measurement may be varied to suit any particular case. This applies to all measurements given hereafter. The outer side and the inner side of the base block are grooved by two slots, into which the end of the splines are fixed and screwed. The slots and the ends of the splines are shaped with a chisel till they fit one another neatly and accurately. The splines are straight pieces of ashwood. They come from broken crutches, all of which are carefully saved for this work. The length of the inside spline is equal to the length of from a point one inch below the ischial tuberosity to the ground. The measurement is taken on the leg which remains and whilst the patient is standing up. In cases of double amputation no exact measurement is required, but the two legs must, of course, be of equal length. The length of the outer spline is from four to six inches greater than the length of the inner. It should be just short of the great trochanter. The strut is a piece of wood of indeterminate length which is fixed between the splines and fastened to each by a screw which goes through the spline and into the end of the strut. Its position is about two inches below the end of the stump. It is the last piece of the leg to be fixed, as its place is only ascertainable after the cast is complete and the stump in it. It takes part of "the harness" to be described hereafter.

G. T. K. Maurice, Colonel A.M.S.,
O.C. Pavilion General Hospital.

*(To be continued.)*

## Figures and Tables

**Figure f1:**